# Comparison of Oral Paracetamol versus Ibuprofen in Premature Infants with Patent Ductus Arteriosus: A Randomized Controlled Trial

**DOI:** 10.1371/journal.pone.0077888

**Published:** 2013-11-04

**Authors:** Dan Dang, Dongxuan Wang, Chuan Zhang, Wenli Zhou, Qi Zhou, Hui Wu

**Affiliations:** 1 Department of Neonatology, The First Hospital of Jilin University, Changchun, China; 2 Department of Ultrasonic Diagnosis, The First Hospital of Jilin University, Changchun, China; 3 Department of Pediatric Surgery, The First Hospital of Jilin University, Changchun, China; Nottingham University, United Kingdom

## Abstract

**Trial Design:**

Oral ibuprofen has demonstrated good effects on symptomatic patent ductus arteriosus (PDA) but with many contraindications and potential side-effects. In the past two years, oral paracetamol administration to several preterm infants with PDA has been reported. Here, a randomized, non-blinded, parallel-controlled and non-inferiority trial was designed to evaluate the efficacy and safety profiles of oral paracetamol to those of standard ibuprofen for PDA closure in premature infants.

**Methods:**

One hundred and sixty infants (gestational age ≤34 weeks) with echocardiographically confirmed PDA were randomly assigned to receive either oral paracetamol (n = 80) or ibuprofen (n = 80). After the initial treatment course in both groups, the need for a second course was determined by echocardiographic evaluation. The main outcome was rate of ductal closure, and secondary outcomes were adverse effects and complications.

**Result:**

The ductus was closed in 65 (81.2%) infants of the paracetamol group compared with 63 (78.8%) of the ibuprofen group. The 95% confidence interval of the difference between these groups was [−0.080,0.128], demonstrating that the effectiveness of paracetamol treatment was not inferior to that of ibuprofen. In fact, the incidence of hyperbilirubinemia or gastrointestinal bleeding in the paracetamol group was significantly lower than that of the ibuprofen group. No significant differences in other clinical side effects or complications were noted.

**Conclusion:**

This comparison of drug efficacy and safety profiles in premature infants with PDA revealed that oral paracetamol was comparable to ibuprofen in terms of the rate of ductal closure and even showed a decreased risk of hyperbilirubinemia or gastrointestinal bleeding. Therefore, paracetamol may be accepted as a first-line drug treatment for PDA in preterm infants.

**Trial Registration:**

ChiCTR.org ChiCTR-TRC-12002177

## Introduction

Patent ductus arteriosus (PDA) in preterm infants is common, with an incidence rate as high as 30% in very low birth weight infants. [1] Persistent PDA in preterm infants can lead to serious clinical consequences, and it is one of the main factors affecting the survival rate of premature children and sequelae incidence. [2], [3] Consequently, clinical intervention to promote ductal closure is necessary.

Currently, the first choice of treatment for PDA is with medication, primarily indomethacin and ibuprofen. The ductal closure rates for these drugs are similar, ranging from approximately 70–85%, [4], [5] but they carry many contraindications and potential side effects.[6]–[11] When drug treatment fails, clinicians may resort to surgical intervention of symptomatic PDA in preterm infants, and the risk of complications from the operation is high. [12], [13] Therefore, a safe and effective alternative drug for the treatment of PDA is urgently needed. Recent studies have shown that paracetamol, a common antipyretic and analgesic drug, can be used to treat PDA in preterm infants with good efficacy and seemingly few side effects. [14] However, it has not been evaluated in a prospective randomized controlled trial. To determine whether oral paracetamol may be used as a first-line drug for PDA in preterm infants, we conducted a randomized, non-blinded, parallel-controlled, non-inferiority trial to compare its efficacy and safety levels to those of ibuprofen. The findings are expected to help extend clinical selections for PDA in preterm infants.

## Patients and Methods

### Patients

The trial was entered in the Chinese Clinical Trial Register (http://www.chictr.org/cn/registration number: ChiCTR-TRC-12002177) and approved by the Hospital Ethics Committee of the First Hospital of Jilin University (No.2012-057). Informed written consent was obtained from parents of the subjects before enrollment. Enrollment criteria were as follows: gestational age ≤34 weeks; postnatal age ≤14 days; echocardiographic diagnosis of hemodynamically significant PDA. Exclusion criteria were: congenital heart disease which required PDA to maintain blood flow; life-threatening infection; recent (within the previous 24 h) intraventricular hemorrhage, grade 3–4; urine output <1 ml per kg per h during the preceding 8 h; serum creatinine >88.4 µmol/L; platelet count of <50×109/L; hyperbilirubinemia requiring exchange transfusion; active necrotizing enterocolitis (NEC) and/or intestinal perforation; liver disfunction. Patients meeting any single exclusion criterion were excluded from the study. The definition of hyperbilirubinaemia is according to Maisels et al. Treatment of jaundice in low birthweight infants [15]. Intestinal bleeding is tendency to bleed as revealed by hematuria, blood in the endotracheal or gastric aspirate or stools, or oozing from puncture sites. Retinopathy (RetCam II, digital imaging system, Clarity Medical Systems, Inc. United States) is based on International Committee for the Classification of Retinopathy of Prematurity [16]. The definition of NEC is on the basis of Bell staging criteria of NEC [17]. Bronchopulmonary dysplasia (BPD) is defined by NICHD (the United States National Institute of Child Health and Human Development ) criteria in 2001 [18].

### Study Design

The protocol for this trial and supporting CONSORT checklist are available as supporting information; see Checklist S1 and Protocol S1. The participants were randomly assigned at a 1∶1 ratio between oral paracetamol and ibuprofen groups by using cards in sealed opaque envelopes. And doctors and nurses were not blind. Infants received oral paracetamol (Acetaminophen suspension drops, Shanghai Johnson & Johnson, 15 ml:1.5 g) at the dose of 15 mg/kg every 6 h for 3 days, or oral ibuprofen (Ibuprofen suspension, Shanghai Johnson & Johnson, 100 ml:2 g) at the initial dose of 10 mg/kg followed by 5 mg/kg after 24 and 48 h. Between doses of oral ibuprofen, infants of ibuprofen group received the same volume of dextrose 5% in water (D5W) as that given for drug administration in the paracetamol group. Whether a subject received a second course of treatment depended on echocardiography evaluation after the first course. If only minor ductal shunting was present after two courses without the need of respiratory support, no further treatment was given. Drug safety factors were assessed daily during the treatment, including 24-h urine output, tendency to bleed, intraventricular hemorrhage (IVH) grade, and serum creatinine and bilirubin levels. An eye examination was conducted 4 weeks after birth. The occurrence of any of the following conditions would prompt the stopping of treatment: renal failure, NEC, IVH grade 3–4, gastrointestinal bleeding.

Main outcome measures were the rates of ductal closure of both drugs after treatment. Every infant was monitored by echocardiography daily during the treatment. Secondary outcomes were the safety of both drugs, including early adverse events (e.g., oliguria, emerging IVH, tendency to bleed, NEC, hyperbilirubinemia, death) and late adverse events, for example BPD, periventricular leukomalacia(PVL), NEC, retinopathy of prematurity (ROP), sepsis, death). The early adverse events were defined as those occurring during and up to 1 week after administration of the drug treatment.

### Statistical Analysis

A study group of 65 patients was needed to detect a difference of at least 20% in the closure rate between the oral paracetamol and ibuprofen groups, assuming a closure rate of 70%[^10^] with oral ibuprofen, with a 95% confidence interval (CI) and a power of 80%. In anticipating that a few patients could be excluded due to various causes during the study, 80 patients in total were enrolled in each group. Interim analyses were performed for main and secondary outcomes at 50% recruitment. The study would be terminated if a difference of 20% in the main outcome or a significant increase in the secondary outcome of the composite variable of death was found. Continuous data were given as means ± SD. Differences between groups were determined by the t test for parametric continuous data, χ^2^ or Fisher`s exact test for categorical data, and Wilcoxon rank-sum test for nonparametric continuous data.

Non-inferiority analysis is a statistical method that is used to determine whether a new drug is non-inferior to a drug of known efficacy. A new drug is considered at least as effective as the known drug if *P*<0.05 or C_L_>-δ (C_L_ is the lower limit of the 95% CI of the difference between two groups; δ is the non-inferiority margin).

SPSS software (version 20.0) was used for all statistical analyses.

## Results

### Baseline Characteristics

**Figure 1:**
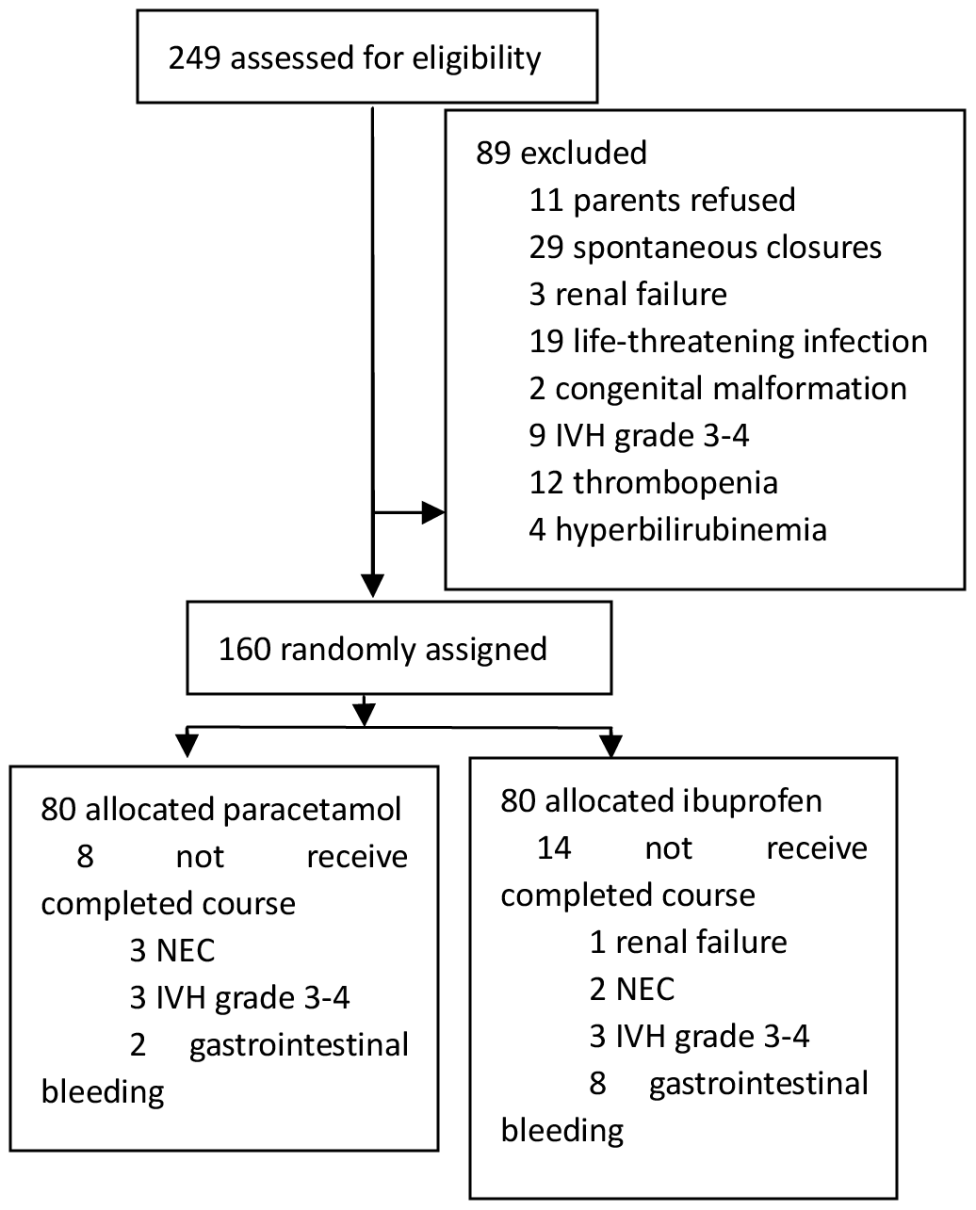
Flow diagram of study infants.

Between May 21, 2012 and March 30, 2013, 1279 preterm infants were treated in our hospital, including 913 infants (71.3%) born at gestational age ≤34 weeks. A total of 249 infants (81.1%) met the enrollment criteria, of whom 89 were excluded (Fig.1). No significant differences in baseline clinical characteristics were observed between the two groups (Table 1).

**Table 1:** Baseline characteristics of study patients.

Characteristic	Ibuprofen group (n = 80)	Paracetamol group (n = 80)	*P* value
Gestational age (week)	30.9±2.2	31.2±1.8	0.474
Birth weight (g)	1531.0±453.5	1591.9±348.6 g	0.342
Gender			0.874
Male	42	41	
female	38	39	
Cesarean birth, n (%)	48(60%)	52(65%)	0.447
PIH, n (%)	33(41.2%)	34(42.5%)	0.873
Antenatal glucocorticoid n (%)	45(56.2%)	47(58.8%)	0.749
Perinatal asphyxia, n (%)	10(12.5%)	11(13.8%)	0.815
Early-onset infection, n (%)	11(13.8%)	10(12.5%)	0.815
Surfactant treatment, n (%)	38(47.5%)	39(48.8%)	0.874
NCPAP, n (%)	52(65.0%)	58(72.5%)	0.306
NSIMV, n (%)	31(38.8%)	29(36.2%)	0.744
SIMV, n (%)	10(12.5%)	12(15.0%)	0.646
IVH grade 1–2, n (%)	11(13.8%)	9(11.3%)	0.633
Mean ductal diameter (mm)	2.36±0.49	2.41±0.44	0.459
Mean max shunt velocity (mm/s)	191.9±30.0	190.8±27.5	0.805
LA/Ao	1.60±0.27	1.67±0.23	0.103

pregnancy induced hypertension syndrome(PIH).

### Efficacy of Treatment

The ductus was closed in 65 infants (81.2%) of the paracetamol group compared with 63 (78.8%) of the ibuprofen group, and there was no significant difference between the two treatments (P = 0.693). Meanwhile, the 95% CI for the difference between the two groups was [−0.080, 0.128]. Thus, the efficacy of paracetamol was non-inferior to the ibuprofen group. After the 1^st^ course of treatment, ductal closure occurred in 45 infants (56.3%) given paracetamol and in 38 infants (47.5%) administered ibuprofen (*P = *0.268). Reopening of the ductus after closure occurred in five infants of the paracetamol group and in six of the ibuprofen group. After continuing to receive the assigned drug treatment, the ductus closed again in four patients of each group (Table 2).

**Table 2:** Efficacy of paracetamol and ibuprofen treatments.

	Paracetamol group (n = 80)	Ibuprofen group (n = 80)	*P* value
Overall closure rate, n (%)	65(81.2%)	63(78.8%)	0.693
Primary closure rate	45(56.3%)	38(47.5%)	0.268
Secondary closure rate	20 (25%)	25(31.3%)	0.379
Reopening after closure	5(7.7%)	6(9.5%)	0.712
Reclosure rate a	4 (80%)	4(66.7%)	0.621
Mean days needed for closure	3.22±0.14	3.71±0.16	0.020

a Ductal closure rate after continuing drug treatment among infants with ductal reopening.

### Safety of Treatment

Eight patients in the paracetamol group and fourteen of the ibuprofen group who did not receive the complete course of treatment were removed from the trial. There were no significant differences between the two groups in the incidence of oliguria, renal failure, NEC, IVH grade and serum creatinine concentration. However, differences in the incidence rates of gastrointestinal bleeding and hyperbilirubinemia between the two groups were significant (*P*<0.05).

There were no significant differences between the two groups in adverse events, including BPD, PVL, NEC, sepsis, ROP and death from one week after treatment onward during the hospitalization period (Table 3).

**Table 3:** Safety profiles of paracetamol and ibuprofen treatments.

	Paracetamol group (n = 80)	Ibuprofen group (n = 80)	*P* value
Early outcomes			
Oliguria	6	9	0.42
Renal failure	0	1	0.32
NEC	3	2	0.65
IVH 1–2	6	7	0.77
IVH 3–4	3	3	1
Hyperbilirubinemia	16	28	0.03
Gastrointestinal bleeding	2	8	0.03
Serum creatinine (mg/dl)	61.62±14.53	62.40±15.24	0.74
Late outcomes			
BPD	4	5	0.73
PVL	6	5	0.59
NEC	3	2	0.65
ROP	7	9	0.60
Sepsis	18	23	0.37
Death	10	12	0.65

## Discussion

Few studies [14], [19]–[21] have been conducted on paracetamol treatment of PDA in preterm newborns to date. In addition, paracetamol was not used as the drug of choice but rather as a supplementary medication in cases where COX inhibitors were ineffective or contraindicated in the majority of several related cases, including the first case [14] reported, in which paracetamol was first used to close ductus arteriosus. As these previous studies lacked sufficient sample sizes for analysis of efficacy and safety-related factors, such as gastrointestinal bleeding, NEC, IVH, hyperbilirubinemia and death, they cannot be used to support paracetamol as a first-line drug for PDA in preterm newborns. Therefore, we conducted a randomized, non-blinded, parallel-controlled, non-inferiority trial in order to compare oral paracetamol and ibuprofen for PDA closure in premature infants. In our study, subjects born at ≤34 weeks of gestation were chosen for enrollment based on the population demographics and clinical needs in China.

We found that oral paracetamol had good efficacy on PDA in preterm infants, and the closure rate of paracetamol was comparable to that of oral ibuprofen. Furthermore, the mean number of days to ductal closure was shorter in the paracetamol group than in the ibuprofen group (3.22±0.14 days vs. 3.71±0.16 days, P = 0.020). Ductal closure in newborns is known to be dependent on increased blood oxygen and decreased vasodilators, including prostaglandin E2 and I2. [2] Prostaglandin synthetase has two different catalytic activities: a cyclooxygenase and a peroxidase. The cyclooxygenase activity catalyzes arachidonic acid to form PGG2, which is then catalyzed by the peroxidase into PGH2. COX inhibitors such as indomethacin and ibuprofen compete with the arachidonic acid substrate for the cyclooxygenase site; thus, the effects of these drugs are influenced by endogenous arachidonic acid levels. [22], [23] Although the precise mechanism of action of paracetamol remains uncertain, it may act at the peroxidase segment of the prostaglandin synthetase to inhibit prostaglandin synthesis. [24], [25] Peroxidase is activated at 10-fold lower peroxide concentrations than that for cyclooxygenase, [25], [26] suggesting that paracetamol can still work well at decreased local peroxide concentrations (e.g., hypoxia). Theoretically, these differences may enable paracetamol to work effectively in the situation where a cyclooxygenase inhibitor is ineffective. Reopening of the ductus after closure was observed in five infants in the paracetamol group and in six infants of the ibuprofen group. After continuing the drug treatment, the ductus closed again in four patients in which it had reopened in each group, suggesting that paracetamol is still effective after the ductal reopening. Regarding the drug safety profile, the incidence rates of gastrointestinal bleeding and hyperbilirubinemia in the paracetamol group were significantly lower than those of the ibuprofen group. Ibuprofen is 99% protein bound, and at higher concentrations, it can be a competitive displacer of bilirubin for albumin binding sites, thereby potentially increasing the risk of hyperbilirubinemia.[27]–[30] In addition, two in vivo studies have demonstrated that ibuprofen treatment results in higher peak levels of total serum bilirubin and longer durations of phototherapy. [31], [32].

This current study has provided several important implications for the clinical treatment of PDA. First, it demonstrated that paracetamol may become the choice drug for PDA in preterm infants. Furthermore, the mean days to closure were shorter in the paracetamol group than in the ibuprofen group (3.22±0.14 days vs. 3.71±0.16 days, P = 0.020), indicating that paracetamol can treat PDA more rapidly compared with ibuprofen, and be better suited for severe cases in which quick relief of symptoms is needed. Finally, the incidence rates of gastrointestinal bleeding and hyperbilirubinemia in the paracetamol group were significantly lower than those of the ibuprofen group. So, paracetamol may be indicated for PDA in preterm infants with hyperbilirubinemia.

Although this study clearly showed that a two-course regimen of paracetamol for premature infants is safe and feasible, some limitations were evident. For example, the results were obtained from the patient population of one medical center. In order to generalize the conclusions, further analysis from a multiple-center, randomized, controlled trial is warranted.

In conclusion, we have demonstrated in this randomized, controlled, non-inferiority trial that paracetamol may be utilized as the drug of choice for PDA in preterm infants with good efficacy and lower risk of gastrointestinal bleeding or hyperbilirubinemia compared with ibuprofen treatment, and is especially suited for those with hyperbilirubinemia. Evidently, paracetamol merits the attention of pediatricians as a new alternative treatment for PDA in preterm newborns.

## Supporting Information

Checklist S1 **CONSORT Checklist.**
(DOC)

Protocol S1 **Trial Protocol.**
(DOC)
