# Supplementary material for: Comparison of Oral Paracetamol versus Ibuprofen in Premature Infants with Patent Ductus Arteriosus: A Randomized Controlled Trial
Source: PLoS One. 2013 Nov 4;8(11):e77888. doi: 10.1371/journal.pone.0077888 (PMC3817181; doi:10.1371/journal.pone.0077888)
Supplement: Protocol S1 — Trial Protocol. (DOC) [file pone.0077888.s002.doc]

Project Title：Comparison of Oral Paracetamol versus Ibuprofen in Premature Infants with Patent Ductus Arteriosus: A Randomized Controlled Trial

Project source：Jilin Department of Health

Institute：the First Hospital of Jilin University

Department: Neonatology

Principal investigator：Wu Hui

Position：Professor，Associated director

Tel: 13134318877

Email：wuhui97@126.com

Date: April 11, 2012

**Bacground**

The ductus arteriosus (DA) is an important vascular connection between the main pulmonary artery and the aorta. After delivery, with the decreasing of vascular resistance, increasing of oxygen content of arterial blood pressure, and reduced secretion of prostaglandin E, ductus arteriosus functionally closed within 10-15 hours after birth.

In premature infants, due to immature arterial wall and less prostaglandin E receptor, their ductus arteriosus failed to close.[1] The incidence of patent ductus arteriosus increases with lower gestional age or birth weight. The reported incidence of an isolated PDA among term infants ranges from 0.04-0.05%, but it ranges between 10-60% in preterm[2,3], with very low birth weight infant(<1500g) 30%, and even higher in extremely low birth weight infant(<1000g)70%.[4, 5]. Significant hemodynamic PDA affects differently depending on gestational age and birth weight. Left to right blood shunt induces an increased risk of pulmonary edema and hemorrhage, bronchopulmonary dysplasia, and a decrease in perfusion and oxygen delivery to end-organs. Persistent PDA in preterm infants can lead to serious clinical consequences and it is one of the main factors affecting the survival rate of premature children and sequelae incidence.[6, 7] Consequently, clinical intervention to promote ductal closure is necessary.

Currently, medication and surgical ligation are valiated effective. Surgical ligation may be performed if the patient remains symptomatic after one or two courses of COX inhibitor or if COX inhibitor treatment is contraindicated. However, pharmacologic therapy remains the preferred initial treatment because ligation is associated with risks of blood pressure fluctuations, respiratory compromise, infection, intraventricular hemorrhage, chylothorax, recurrent laryngeal nerve paralysis, BPD, and death.[8, 9] The ductal closure rates for these drugs, primarily indometacin and ibuprofen are similar, ranging from approximately 70–85%, but they carry many contraindications and potential side effects.[10-15] Oral indomethacin casues more than intravenous indomethacin, the very drug that is uses in United States for PDA treatment, the incidence of gastrointestinal complications, such as necrotizing enterocolitis, intestinal perforation. However, a large number of RCT show that ibuprofen can have the same effect.[16-24] Oral ibuprofen can cause feeding intolerance, gastrointestinal bleeding, gastrointestinal perforation even necrotizing enterocolitis[25, 26] There is no intravenous indomethacin or ibuprofen in China, therefore, a safe and effective alternative drug for the treatment of PDA is urgently needed.

Paracentomal, a common antipyretic and analgesic drug, inhibit prostaglandin E synthesis in its way different from non-steroidal anti-inflammatory drugs. Although the precise mechanism of action of paracetamol remains uncertain, it may act at the peroxidase segment of the prostaglandin synthetase to inhibit prostaglandin synthesis. Oral formulations suitable for newborn application is easily access in China. Recent studies have shown that paracetamol can be used to treat PDA in preterm infants with good efficacy and seemingly few side effects.[27] However, it has not been evaluated in a prospective, randomized, controlled trial.

We conducted a randomized, single-blind, parallel-controlled, non-inferiority trial that compare the efficacy and safety levels between paracentomal with ibuprofen. The findings are expected to help extend clinical selections for PDA in preterm infants.

**References**

1. ER Hermes-DeSantis1. RI Clyman. Patent ductus arteriosus: pathophysiology and management.Journal of Perinatology (2006) 26, S14–S18.

2. Hamid Amoozgar. Mohammad Ghodstehrani. Narjes Pishva. Oral Ibuprofen and Ductus Arteriosus Closure in Full-Term Neonates: A Prospective Case–Control Study.Pediatr Cardiol (2010) 31:40–43.

3. Dani C. New therapeutic strategies for the treatment of patent ductus arteriosus. J Matern Fetal Neonatal Med 2011;24: S32–S33.

4. Federico Schena, elena ciarmoli, Fabio Mosca. Patent ductus arteriosus: wait and see? The Journal of Maternal-Fetal and Neonatal Medicine, 2011; 24(S(3)): 2–4.

5. Koch J, Hensley G, Roy L, Brown S, Ramaciotti C, Rosenfeld CR. Prevalence of

spontaneous closure of the ductus arteriosus in neonates at a birth weight of 1000 grams or less. Pediatrics 2006;117:1113–1121.

6. Shannon E. G. Hamrick, and Georg Hansmann. Patent Ductus Arteriosus of the Preterm Infant. Pediatrics 2010;125;1020

7. Giovanbattista capozzi, Giuseppe Santoro. Patent ductus arteriosus: patho-physiology, hemodynamic effects and clinical complications. The Journal of Maternal-Fetal and Neonatal Medicine, 2011; 24(S(1)): 15-16.

8. Kabra NS, Schmidt B, Roberts RS, Doyle LW, Papile L, Fanaroff A; Trial of Indomethacin Prophylaxis in Preterms Investigators. Neurosensory Impairment after Surgical Closure of Patent Ductus Arteriosus in Extremely Low Birth Weight Infants: Results from the Trial of Indomethacin Prophylaxis in Preterms. J Pediatr 2007;150:229-34.

9. Malviya MN, Ohlsson A, Shah SS. Surgical versus medical treatment with cyclooxygenase inhibitors for symptomatic patent ductus arteriosus in preterm infants. Cochrane Database of Systematic Reviews 2008, Issue 1. Art. No.: CD003951. DOI: 10.1002/14651858.CD003951.pub2.

10. J. V. Aranda, FRCPC, FAAP,Ronald Thomas. Intravenous Ibuprofen for Preterm Newborns. NeoReviews 2005;6;e516-e523.

11. R Rao, K Bryowsky, J Mao, D Bunton, C McPherson, A Mathur. Gastrointestinal complications associated with ibuprofen therapy for patent ductus arteriosus. Journal of Perinatology (2011) 31, 465–470.

12. Alla Kushnir, Joaquim MB Pinheiro.Comparison of renal effects of ibuprofen versus indomethacin during treatment of patent ductus arteriosus in contiguous historical cohorts. BMC Clinical Pharmacology 2011, 11:8.

13. Nidhi A. Shah, Nancy K. Hills, Nahid Waleh, Donald McCurnin, Steven Seidner, Sylvain Chemtob, and Ronald Clyman, Relationship between Circulating Platelet Counts and Ductus Arteriosus Patency after Indomethacin Treatment. J Pediatr 2011;158:919-23.

14. Ohlsson A, Walia R, Shah SS. Ibuprofen for the treatment of patent ductus arteriosus in preterm and/or low birth weight infants. Cochrane Database of Systematic Reviews 2010, Issue 4. Art. No.: CD003481. DOI: 10.1002/14651858.CD003481.pub4.

15. Lynda Adrouche-Amrani, Robert S Green, Karen M Gluck and Jing Lin. Failure of a repeat course of cyclooxygenase inhibitor to close a PDA is a risk factor for developing chronic lung disease in ELBW infant. BMC Pediatrics 2012 12:10.

16. Aly H, Lotfy W, Badrawi N, et al. Oral Ibuprofen and ductus arteriosus in premature infants: A randomized pilot study. Am J Perinatol. 2007;24:267–270.

17. Fakhraee SH, Badiee Z, Mojtahedzadeh S, et al. Comparison of oral ibuprofen and indomethacin therapy for patent ductus arteriosus in preterm infants. Zhongguo Dang Dai Er Ke Za Zhi. 2007;9:399–403.

18. Cherif A, Jabnoun S, Khrouf N. Oral ibuprofen in early curative closure of patent ductus arteriosus in very premature infants. Am J Perinatol. 2007;24:339–345.

19. Pourarian S, Pishva N, Madani A, Rastegari M. Comparison of oral ibuprofen and indomethacin on closure of patent ductus arteriosus in preterm infants. East Mediterr Health J. 2008;14:360–365.

20. Cherif A, Khrouf N, Jabnoun S, et al. Randomized pilot study comparing oral ibuprofen with intravenous ibuprofen in very low birth weight infants with patent ductus arteriosus. Pediatrics. 2008;122:e1256–e1261.

21. Manjunath S, Hariram M. Study of efficacy of oral ibuprofen in preterm neonates with PDA and impact on short term clinical variables. Early Hum Dev. 2008;84(Suppl):S154.

22. Salama H, Alsisi A, Al-Rifai H, et al. A randomized controlled trial on the use of oral ibuprofen to close patent ductus arteriosus in premature infants. J Neonatal Perinatal Med. 2008;1:153–158.

23. Erdeve O, Sarici SU, Sari E, Gok F. Oral-ibuprofen-induced acute renal failure in a preterm infant. Pediatr Nephrol. 2008;23:1565–1567.

24. Erdeve O, Gokmen T, Altug N, Dilmen U. Oral versus intravenous ibuprofen: Which is better in closure of patent ductus arteriosus? Pediatrics. 2009;123:e763.

25. Jean-Bernard Gouyon, Yves Kibleur. Efficacy and Tolerability of Enteral Formulations of Ibuprofen in the Treatment of Patent Ductus Arteriosus in Preterm Infants. Clin Ther. 2010; 32:1740–1748.

26. Erdeve O, Gokmen T, Altug N, Dilmen U. Oral versus intravenous ibuprofen: which is better in closure of patent ductus arteriosus? Pediatrics. 2009 Apr;123(4):e763.

27. Cathy Hammerman, Alona Bin-Nun, Einat Markovitch, Michael S. Schimmel, Michael Kaplan, MBChB, Daniel Fink. Ductal Closure With Paracetamol: A Surprising New Approach to Patent Ductus Arteriosus Treatment. Pediatrics 2011;128;e1618.

**Protocol**

**1.Design**

A randomized, single-blind, parallel-controlled, non-inferiority trial

**2.Inclusion criteria**

1)Gestational age≤34W

2) Postnatal age less than 2 weeks

3)Clinical manifestations or Hemodynamic significant

a. Clinical manifestations （any one of the follows） hyperdynamic precordium; heart rate >165 BPM; systolic murmur; pulmonary edema; metabolic acidosis; bounding pulses; hepatosplenomegaly.

b. Hemodynamic significant（any one of the follows）① a left atrium to aortic root diameter ratio of ≥1.4 in the parasternal long-axis view; ② a DA diameter of ≥1.4 mm/kg body weight; ③ left ventricular enlargement; ④ Holodiastolic flow reversal in the descending aorta

**3.Exclusion criteria**

1)Congenital heart disease which need PDA to maintain blood flow

2)Life-threatening infection

3)Recent (within the previous 24 hours) intraventricular hemorrhage Grade 3-4

4)Urine output <1 ml per kilogram per hour during the preceding 8 hours

5)Serum creatinine ＞88.4μmol/L

6) Platelet count of <50X109/L.

7) Hyperbilirubinaemia which need exchange transfusion or liver disfunction

8) Active NEC and/or intestinal perforation

**4.Termination standard:**

Test termination refers to that clinical trials stop before the end, for the purpose to protect the rights and interests of subjects and to ensure the test quality.

1)Sever safety issues occur during the trals;

2)Intervention drug was proved poor or no efficacy;

3)Serious medical errors in protocol that makes estimation impossible;

4) Administration reasons;

5)Withdraw by State food and drug administration。

**5.Drop out standard**

Subjects who sign the informed consent and donot complete the prescribed observation period regardless of when and where to exit, are called drop out.

In this case, try to contract with the subjects, ask the reason, record the last time of medication, and complete the assessment. Corresponding treatment measures should be taken when allergic or adverse reactions occur.

**6. Steps of subjects withdrawing from the study**

The enrollees can exit the experiment in the following cases, and if probable, the outcomes should be recorded detailedly.

1) withdrawal determined by the researcher: the experiment should be quit if any one of the following conditions occur.

a. Enrollees found in the study course who do not meet the inclusion criteria or do follow the exclusion standards.

b. Enrollees who get other irrelevant diseases that could affect the assessment on the efficiency and safety of the drug during the test.

c. Patients who receive inhibitors of prostaglandin E that influences the assessment on the drug.

d. Patients who do not strictly take standard medication.

2) withdrawal determined by the infants’ parents ( such subjects should be included in the safety analysis).

**7. Intervention:**

Computer-generated randomization table, the drug type of treatment received by each subject generated at a 1:1 ratio.

(1) Treatment group receive oral paracetemol at the a doses of 15 mg/kg every 6 h for 3 days.

(2) Control group receive oral ibuprofen at the initial dose of 10 mg/kg followed by 5 mg/kg after 24 and 48 h. Between doses of oral ibuprofen, infants of ibuprofen group received the same volume of dextrose 5% in water (D5W) as that given for drug administration in the treatment group.

**8.Outcomes**

1)Main outcome measure:The rates of ductal closure of both drugs after treatment.

2)Secondary outcomes :

Early adverse events: oliguria, emerging IVH, tendency to bleed, NEC, hyperbilirubinemia, death;

Late adverse events: [bronchopulmonary dysplasia](http://www.iciba.com/bronchopulmonary_dysplasia)（BPD), periventricular leukomalacia(PVL) , NEC, retinopathy of prematurity （ROP）,sepsis, death

**9.Feasibility Analysis**

1) The number of patients enrolled for this project can be ensured at the department of neonatology in the First Hospital of Jilin University. There are 150 beds in our department. Because our department is the sole neonatal retrieval service for the province, we receive often the most seriously ill newborn infant. 4012 admissions including 1541 prematures were treated here in 2011.

2) There are excellent medical facilities for the diagnosis ant treatment of PDA in preterm infants, ensuring that the test is performed successfully.

3)Staff in this research group are excellent and experienced. Professor Wu Hui, the director in the group, who once received systematic clinical training(fellowship) at the Foothill medical center in University of Calgary in Canada, is accomplished in clinical randomized controlled trials and has explored in depth the PDA in prematures. Meanwhile, Dongxuan Wang, an expert majore in cardiovascular ultrasound，can ensure an objective and scientific test.

4)Our project was entered in the Chinese Clinical Trial Register ([Http://www.chictr.org](http://www.chictr.org/)) and would be overlooked in the course of the test.

**10.Route**

1.Registate in the Chinese Clinical Trial Register (http://www.chictr.org/cn/ registration number: ChiCTR-TRC-12002177) and approved by the Hospital Ethics Committee.

2. Collect Patients according to inclusion and exclusion criteria.

3.Informed written consent was obtained from parents of the subjects before enrollment.

4. Allocation

1)Paracentomal group：oral paracetemol at the a doses of 15 mg/kg every 6 h for 3 days.

2) Ibuprofen group: oral ibuprofen at the initial dose of 10 mg/kg followed by 5 mg/kg after 24 and 48 h. Between doses of oral ibuprofen, infants of ibuprofen group received the same volume of dextrose 5% in water (D5W) as that given for drug administration in the paracetamol group

5.Record the baseline and evaluation values of infants.

6. Statistic analysis and compose articles.

**11.Innovation**

Efficacy of intravenous or oral ibuprofen and indomethacin is valiated worldwide,yet there is still no randomized controlle trial using paracentomal for PDA treatment. Due to unavaliabe of intravenous ibuprofen or indomethacin, side effect of oral ibuprofen limits its clinical use. We conducted a randomized controll trial to compare its efficacy and safety levels to those of ibuprofen. The findings are expected to help extend clinical selections for PDA in preterm infants.

**12.Project duration**

2012-5-1 to 2012-11-1: Design study, set up inclusion, exclusion criteria, random scheme, and calculate the number of cases. Implementation of the project. Anticipate target: Complete 100 cases

2012-11-1 to 2013-5-1: Anticipate target：Complete 60 cases. Compose articles
